# Deep-ocean dissolved organic matter reactivity along the Mediterranean Sea: does size matter?

**DOI:** 10.1038/s41598-017-05941-6

**Published:** 2017-07-18

**Authors:** Alba María Martínez-Pérez, Xosé Antón Álvarez-Salgado, Javier Arístegui, Mar Nieto-Cid

**Affiliations:** 1 Consejo Superior de Investigaciones Científicas - Instituto de Investigacións Mariñas (CSIC-IIM), Vigo, Spain; 2 Instituto de Oceanografía y Cambio Global (IOCAG), Universidad de Las Palmas de Gran Canaria, 35017 Las Palmas de Gran, Canaria Spain

## Abstract

Despite of the major role ascribed to marine dissolved organic matter (DOM) in the global carbon cycle, the reactivity of this pool in the dark ocean is still poorly understood. Present hypotheses, posed within the size-reactivity continuum (SRC) and the microbial carbon pump (MCP) conceptual frameworks, need further empirical support. Here, we provide field evidence of the soundness of the SRC model. We sampled the high salinity core-of-flow of the Levantine Intermediate Water along its westward route through the entire Mediterranean Sea. At selected sites, DOM was size-fractionated in apparent high (aHMW) and low (aLMW) molecular weight fractions using an efficient ultrafiltration cell. A percentage decline of the aHMW DOM from 68–76% to 40–55% was observed from the Levantine Sea to the Strait of Gibraltar in parallel with increasing apparent oxygen utilization (AOU). DOM mineralization accounted for 30 ± 3% of the AOU, being the aHMW fraction solely responsible for this consumption, verifying the SRC model in the field. We also demonstrate that, in parallel to this aHMW DOM consumption, fluorescent humic-like substances accumulate in both fractions and protein-like substances decline in the aLMW fraction, thus indicating that not only size matters and providing field support to the MCP model.

## Introduction

Most of the 662 Pg C of dissolved organic matter (DOM) accumulated in the oceans is resistant to microbial degradation and, therefore, stored for hundreds to thousands of years^1, 2^. The mechanisms behind this long-term storage of carbon are still poorly understood^3^. The microbial carbon pump (MCP) concept has emerged in recent years as one of the most plausible mechanisms to explain this storage. Within the MCP framework, refractory DOM is originated as a by-product of the microbial mineralization of bioavailable organic matter^4, 5^.

Closely related with the MCP concept is the size-reactivity continuum (SRC) hypothesis to explain marine DOM reactivity^6^. According to this assumption, initially based on microbial degradation experiments^7, 8^, changes in the bioavailability of DOM would be explained by varying proportions of more labile high molecular weight (HMW) DOM compared with slower degrading low molecular weight (LMW) DOM. Earlier works showing patterns, compositions and concentrations of major DOM biochemical compounds (carbohydrates and amino acids) in the ocean provided independent validation of the relative reactivity of different size classes of organic matter^9–11^. Posterior field studies supported the SRC hypothesis as they found higher Δ^14^C values in the HMW DOC fraction compared to the LMW DOC fraction^12, 13^. Lastly, it has been recently reported a significant dissolved organic matter size-age-composition relationship that is also consistent with the SRC model^14–16^. These studies suggest microbial degradation as the primary source of recalcitrant DOM to the deep ocean, in agreement with the MCP conceptual model. Nevertheless, other works based on bacterial degradation experiments have found discrepancies with the SRC model^17, 18^, as they observed that LMW DOM is biologically more reactive than HMW DOM.

Focusing only on the colored fraction of DOM (CDOM) a different picture may arise, facing the SRC hypothesis. It is well established that fluorescence spectroscopy measurements at specific wavelength pairs are due to refractory humic-like substances and to bioavailable protein-like compounds^19, 20^. Microbial cultures have demonstrated consumption of the protein-like and production of the humic-like DOM in a daily to yearly basis^21–23^. The generation of humic-like substances implies the formation of structurally complex molecules that, apparently, challenges the SRC postulates.

Here, we assess the validity of the SRC model *in situ*, by following the course of two size fractions of DOM along the shallow overturning circulation cell of the Mediterranean Sea^24, 25^. We chose this region because its high temperatures boost biogeochemical rates, resulting in shorter spatial and time scales, acting as a natural laboratory to explore processes happening at a global extent. The shallow branch of the Mediterranean overturning circulation drives the Levantine intermediate water (LIW), which is formed by convection in the northern part of the Levantine basin. LIW is found along the whole Mediterranean Sea between 200 m depth in the eastern and 500 m depth in the western basin, displaying the maximum salinity of all Mediterranean water masses^26^. According to the SRC hypothesis, the LIW in the Levantine basin (close to its formation site) should transport a higher concentration of DOM with a higher average molecular weight. This DOM would be progressively consumed within the overturning cell in such a way that the LIW leaving the Strait of Gibraltar would transport less DOM with a lower average molecular weight. Regarding the optical properties of DOM, at the formation site we would expect enrichment in bioavailable protein-like substances and depletion in refractory humic-like compounds. Conversely, as the LIW displaces westwards, the proportions of protein- and humic-like substances should reverse.

## Results and Discussion

**Figure 1:**
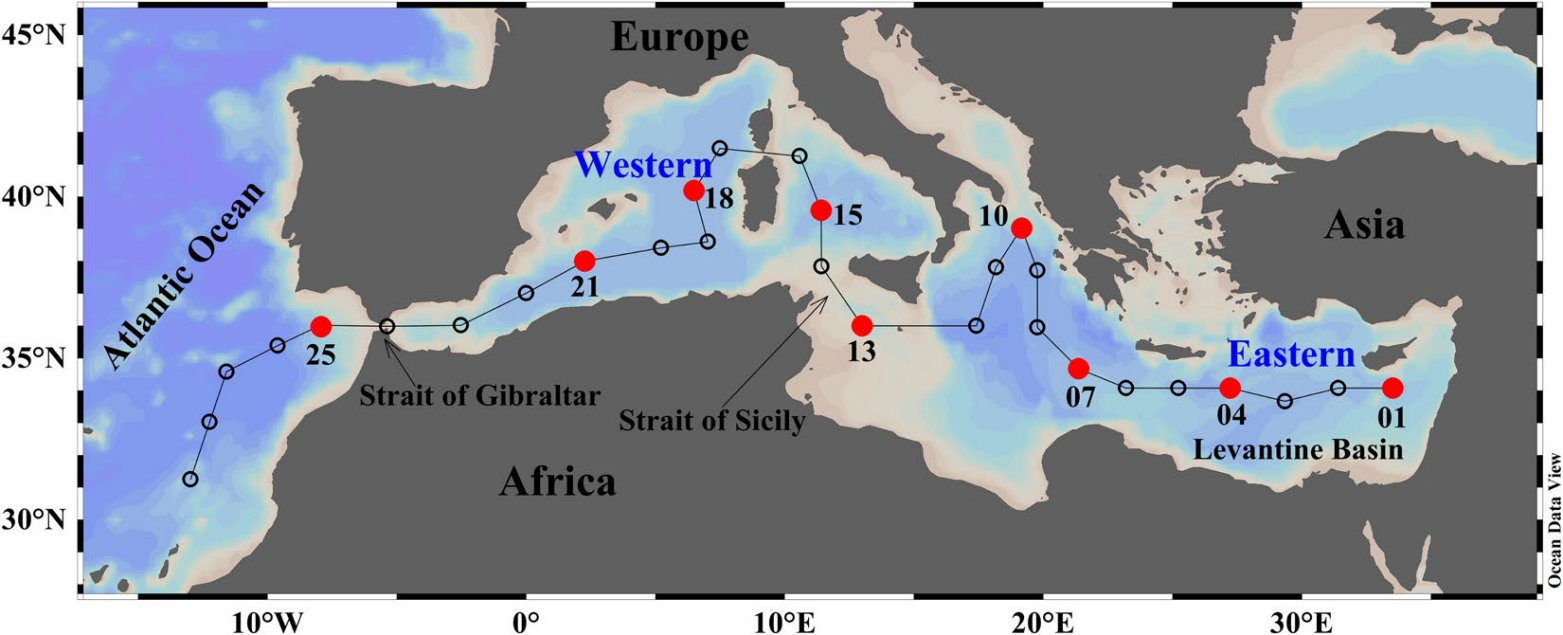
Map of the study area and sampling stations during the HOTMIX cruise. Open circles indicate all stations sampled during the cruise and solid red circles represent the stations where samples for DOM size-fractionation were taken. Figure created using Ocean Data View (Schlitzer, R., Ocean Data View, odv.awi.de, 2017).

Nine stations were occupied along the Mediterranean Sea (Fig. 1)^27^ for DOM sampling at different depths covering the whole water column. The deep chlorophyll maximum (DCM), Levantine Intermediate Water (LIW) and the Eastern (EMDW) and Western Mediterranean Deep Water (WMDW) levels were sampled (for more details see Methods; Fig. S1). These DOM samples were size-fractionated using a 1 kDa cut-off membrane (Millipore, PLAC 150 mm), and the dissolved organic carbon (DOC) and fluorescence intensity (FDOM) of all fractions were determined (see Methods). On a carbon basis, the mass balance for the size-fractionation process presented an error lower than 10% for all the samples. The high recovery of our low-volume ultrafiltration cell (50–80%) was mainly due to the low concentration factor (CF) of just 4 used in this study. This cell allowed us to separate the size fractions in less than 5 hours in such a way that several samples can be processed within the same day (see Methods). On the contrary, standard ultrafiltration methodologies for large sample volumes, isolate about 30–55% of the marine DOM^28, 29^, applying CFs ranging from about 12 to about 500, depending on the purpose of the ultrafiltration (%HMW quantification^28, 30^ *versus* isolation^13^). Ultrafiltration using higher CFs is more time consuming, which is not compatible with our oceanographic approach. Given that our separation method is not comparable with the classical approaches in using ultrafiltration methods, we refer hereinafter to the fraction <1 kDa as apparent LMW DOM (aLMW DOM) and the fraction >1 kDa as apparent HMW DOM (aHMW DOM). Furthermore, due to the ultrafiltration system and the CF used in this study, our aHMW fraction may include up to 25% of low molecular weight molecules (see Methods).

### Testing the size-reactivity continuum hypothesis

DOC and the carbon-specific fluorescence of humic-like (peak C*) and protein-like (peak T*) substances for the different sampling depths are represented against the apparent oxygen utilisation (AOU; see Methods) in Fig. S2. DCM or deep water samples showed narrow AOU variations through the cruise track (mean ± SD; 4.5 ± 3.1 and 64.4 ± 5.2 µmol kg^−1^, respectively; blue rectangle boxes in Fig. S2). Recently ventilated DCM waters presented very low AOU values, whereas aged deep waters exhibited significantly higher values. Instead, samples collected at the intermediate salinity maximum layer displayed a wide AOU range (mean ± SD; 50.2 ± 30.3 µmol kg^−1^; red rectangle boxes in Fig. S2) revealing an increasing trend from the Levantine basin (water mass formation area) to the Strait of Gibraltar (Figs S1 and S2). For the sake of the interest of our study, we focused on samples with the highest proportion of LIW, which is the single water mass crossing the entire Mediterranean Sea. This allowed us to observe changes in DOM size-fractionation, as this intermediate water was ageing along their westward route from the Levantine basin to the Atlantic Ocean^25^.

**Figure 2:**
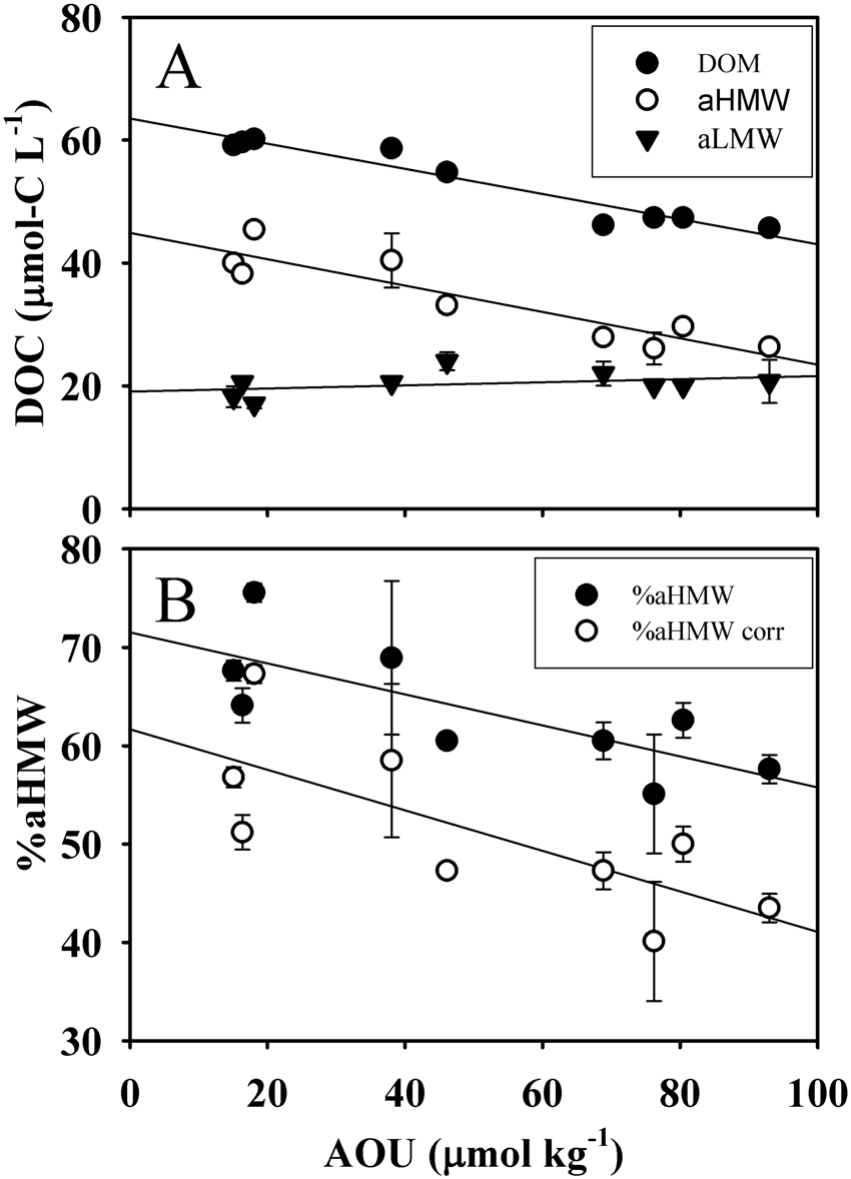
DOM size-fractionation with apparent oxygen utilization on a carbon basis. (**A**) size-fractionated DOC where DOM (solid black circles), aHMW (open circles) and aLMW (solid black triangles) represent the bulk DOC, the apparent high molecular weight fraction and the apparent low molecular weight fraction, respectively. (**B**) aHMW percentage (solid black circles) and corrected aHMW percentage (open circles) with respect to apparent oxygen utilization (AOU) of samples collected along the core-of-flow of the Levantine Intermediate Water. Error bars represent standard errors.

**Figure 3:**
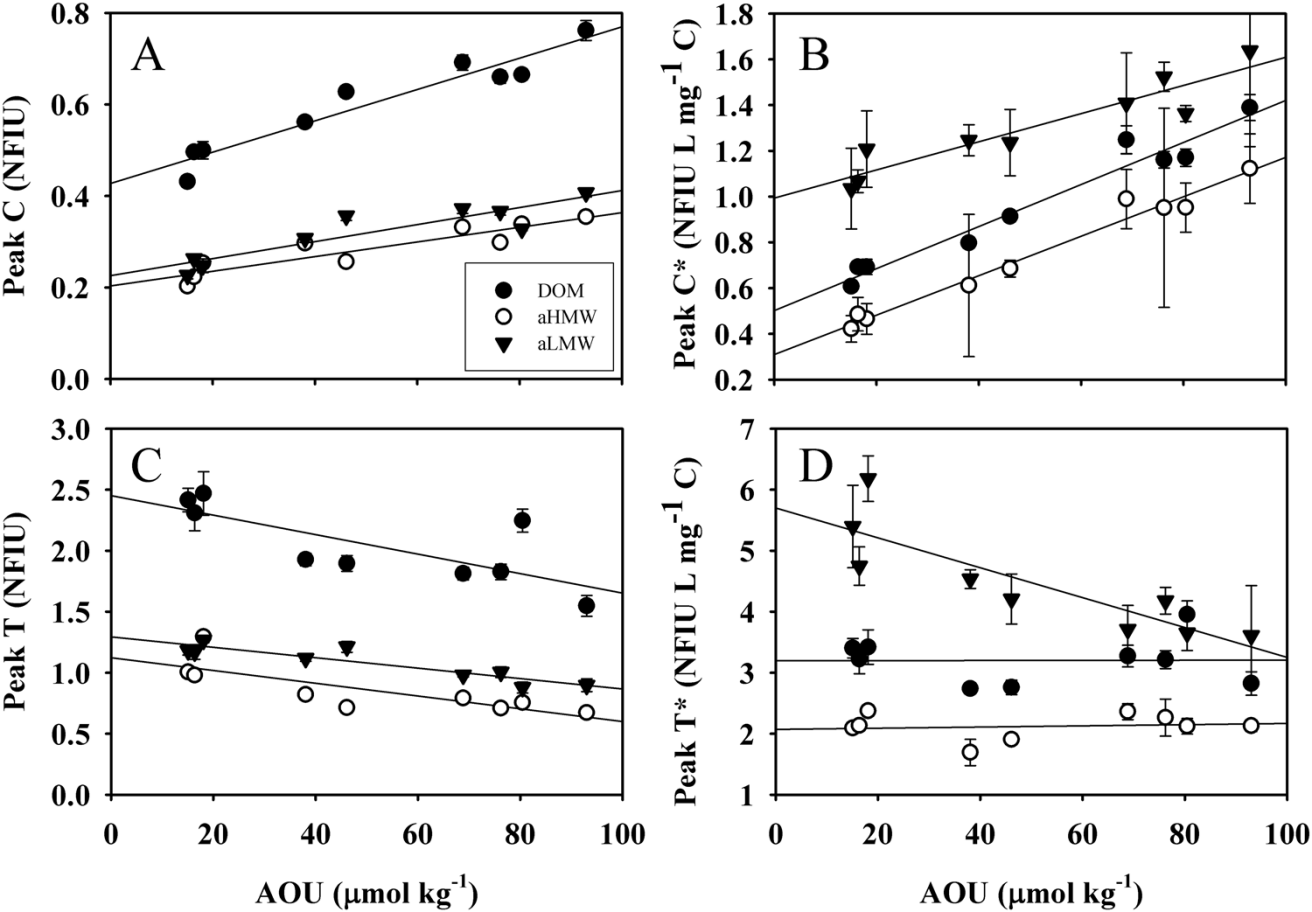
DOM size-fractionation with apparent oxygen utilization on a fluorescence basis. DOM (solid black circles), aHMW (open circles) and aLMW (solid black triangles) represent the bulk DOM, the apparent high molecular weight fraction and the apparent low molecular weight fractions, respectively, of samples collected along the core-of-flow of the Levantine Intermediate Water. (**A**) Humic-like fluorescence (peak C), (**B**) humic-like fluorescence per carbon unit (peak C*), (**C**) protein-like fluorescence (peak T) and (**D**) protein-like fluorescence per carbon unit (peak T*) with respect to apparent oxygen utilization (AOU). Error bars represent standard errors.

The DOM size-fractionation at the LIW level is illustrated in more detail in Figs 2 and 3. A significant decrease of DOC with increasing AOU was detected for the bulk DOM and the aHMW fraction (Fig. 2A), revealing that larger organic compounds are preferably consumed during mineralization processes. However, the aLMW DOC concentration showed a slight increase with AOU, although it was not significant. Note that although up to 25% of the aHMW fraction corresponds with low molecular weight molecules, the changes observed cannot be due to this smaller DOM since, as observed in Fig. 2A, the LMW fraction did not undergo any decay with AOU. Thus, we can infer that larger molecules are more bioreactive. Taking into account the slopes of the linear regressions (Fig. 2A, Table 1) and converting them into carbon equivalents using the canonical Redfield -O_2_/C ratio of 1.4^31^ we obtained that 30 ± 3% of the oxygen utilization at the LIW was due to the DOM decomposition. This result is in agreement with the value reported for the intermediate and deep waters of the Eastern Mediterranean Sea (27 ± 18%)^32^, but slightly lower than the 38% reported for the LIW^33^. Our values are higher than those found in the global ocean (10–20%)^34^ and highlight the relevance of the DOC pool for oxygen consumption in the mesopelagic layers of the Mediterranean Sea. In addition, the partition of DOC into aHMW and aLMW allowed to establish, for the first time, the contribution of each apparent fraction to the overall mineralization. As observed in Fig. 2A, the aLMW DOC did not contribute to the oxygen consumption, while the aHMW DOC accounted for as much as the bulk DOC (32 ± 6%). Therefore, all the DOC mineralization was due exclusively to the aHMW DOC, a fact that validates the SRC hypothesis^6^ using the shallow overturning cell of the Mediterranean Sea as an *in situ* incubator. Note that this result is valid for the bulk DOC but does not necessarily apply to the myriads of individual compounds that constitute this pool. Furthermore, considering the intercepts of the three regression equations (being all significant, p < 0.001; Table 1), we can estimate the DOC concentration at the time of water mass formation (when AOU should be null). It results in 63 ± 1 µmol-C L^−1^, of which 45 ± 2 µmol-C L^−1^ corresponds to aHMW DOC and 19 ± 1 µmol-C L^−1^ to aLMW DOC. If we discount a 25% due to molecules smaller than 1 kDa that are present in the aHMW fraction, the corrected partition turns to be 39 µmol-C L^−1^ of aHMW DOC and 25 µmol-C L^−1^ of aLMW DOC. The DOC concentration at the formation time for the bulk DOC is comparable with the previously reported value for the LIW (67 ± 1 µmol-C L^−1^)^33^. The evolution of the percentage of aHMW DOM (%aHMW, on a carbon basis) with the AOU along the LIW transit from its formation site (Fig. 2B) shows a significant inverse relationship (r^2^ = 0.58, p < 0.05). This relationship is independent of considering (black circles in Fig. 2B) or not (white circles in Fig. 2B) the 25% of LMW molecules contained in the aHMW DOM fraction. The slopes of both linear regressions are not significantly different. This observation supports the view that aHMW molecules are preferentially consumed in the intermediate waters of the Mediterranean Sea simultaneously to water mass ageing, and therefore large DOM would be more bioreactive than the smaller counterpart. It also corroborates that, although our size fractionation system does not allow obtaining a 100% pure aHMW DOM fraction, our conclusions are not influenced by this fact. Moreover, our findings are in agreement with the size-age-composition relationships of DOM reported in recent studies^14–16^.

**Table 1:** Linear regressions (model II) between DOC and FDOM indices (y-variable) and apparent oxygen utilization (x-variable) obtained from bulk DOM and apparent HMW (aHMW) and LMW (aLMW) fractions from samples collected at the salinity maximum (LIW). n = 9, m = slope, b = intercept, SE = Standard error and NS = Non significant. *, **, *** correspond with p < 0.05, p < 0.01 and p < 0.001, respectively. DOM <0.7 µm, aHMW <0.7 µm and >1 kDa and aLMW <1 kDa.

Fraction	y-variable	m ± SE	b ± SE	R^2^
DOM	DOC	−21 ± 2 × 10^−2^***	63 ± 1***	0.93
aHMW	DOC	−23 ± 4 × 10^−2^***	45 ± 2***	0.84
aLMW	DOC	7 ± 5 × 10^−2NS^	19 ± 1***	0.15
DOM	Peak C	35 ± 4 × 10^−4^***	0.43 ± 0.02***	0.92
aHMW	Peak C	18 ± 3 × 10^−4^***	0.20 ± 0.02***	0.83
aLMW	Peak C	21 ± 3 × 10^−4^***	0.23 ± 0.02***	0.84
DOM	Peak C*	9 ± 1 × 10^−3^***	0.50 ± 0.04***	0.96
aHMW	Peak C *	87 ± 5 × 10^−4^***	0.31 ± 0.03***	0.97
aLMW	Peak C*	6 ± 1 × 10^−3^***	1.00 ± 0.05***	0.87
DOM	Peak T	−10 ± 4 × 10^−3^*	2.4 ± 0.1***	0.58
aHMW	Peak T	−6 ± 1 × 10^−3^*	1.1 ± 0.1***	0.63
aLMW	Peak T	−4 ± 1 × 10^−3^***	1.29 ± 0.04***	0.83
DOM	Peak T*	0.01 ± 0.6^NS^	3.2 ± 0.3***	0.00
aHMW	Peak T *	7 ± 20 × 10^−3 NS^	2.1 ± 0.1***	0.02
aLMW	Peak T*	−28 ± 7 × 10^−3^**	5.7 ± 0.3***	0.73

Taking into account that the renewal time of the LIW in the entire Mediterranean Sea is about 13 years^35^ and that DOC decreases by 18 µmol-C L^−1^ from the formation area of the LIW (DOC = 63 µmol L^−1^) to the Strait of Gibraltar (DOC = 45 µmol L^−1^), a DOC removal rate of 1.38 µmol-C L^–1^ yr^−1^ is obtained. This rate is lower than the previously reported rate of 2.2 µmol-C kg^−1^ yr^−1^ ^36, 37^ because the latter was an estimate for the eastern Mediterranean Sea including the Tyrrhenian Sea, whereas our DOC removal rate includes the whole Mediterranean Sea. The calculated DOC removal rate and its corresponding lifetime indicate that the aHMW DOC consumed along the core-of-flow of the LIW is a mixture of semi-labile (lifetime, 1.5 years) and semi-refractory (lifetime, 20 years) DOC according to Hansell’s classification^38^.

It could be argued that the DOC-AOU relationship (Fig. 2A) is distorted by the sinking flux of biogenic particulate organic carbon (POC). However, in the ultra-oligotrophic eastern basin, where the core-of-flow of the LIW is shallower (200–300 m), the vertical flux of POC is minor compared with the injection of DOC in the water mass formation area of the LIW^39, 40^. In the western basin, although the vertical flux of POC is dominant, the amount reaching the core-of-flow of the LIW at 350–400 m presents high turnover rates. As POC consists mainly on the labile products of synthesis and early degradation of plankton^41^, the POC reaching the intermediate waters will be degraded in time scales of hours to weeks^42^. Therefore, the observed DOC-AOU relationship along the core-of-flow of the LIW would be essentially driven by the consumption of semi-refractory HMW DOC than of labile sinking POC.

### Testing the microbial carbon pump hypothesis

There is little information on the size distribution of coloured DOM (CDOM) in ocean waters^43–45^. Here we observed that the aLMW fraction hosts more humic- and protein-like fluorescence intensity per carbon unit than the aHMW DOM fraction (Fig. 3B,D). Particularly, for the protein-like fluorescence (peak T) the contribution is higher in the less aged waters (low AOU), while for the humic-like fluorescence (peak C) the two DOM fractions presented a significant increase with AOU (Fig. 3A,B). This is indicative of the production of humic-like fluorescence per unit of carbon in parallel to the general trend of DOC and aHMW DOC consumption. All together, these observations support the MCP hypothesis, which postulates that refractory DOM is produced during the mineralization of bioavailable organic matter, either dissolved or in suspended and sinking particles^5^. Specifically, the generation rate of humic-like substances was 30% higher in the HMW DOC than in the LMW DOC (Table 1; Fig. 3B) due to DOC dynamics: aHMW DOC was consumed while LMW DOC remained constant. On the other hand, peak T exhibited a significant decrease in both DOM fractions with AOU (Fig. 3C), indicative of the utilization of this type of compounds during mineralization processes. This finding suggests that small organic compounds related to protein-like molecules were also degraded when intermediate waters aged. Changes of peak T in the LMW fraction were not reflected in the bulk DOC due to the lower sensitivity of the DOC analytical technique compared with fluorescence spectroscopy. Therefore, the LMW fraction also contains some bioreactive molecules although the bulk fraction is essentially refractory. For the aHMW DOC and the bulk DOC we observed that the rate of protein-like substances consumption was equal to the rate of DOC consumption (as the horizontal lines in Fig. 3D suggest). These results indicate that the overall behaviour of the bulk DOC pool does not necessarily match the specific response of particular compounds or compound groups as the fluorophores studied here, and reconcile the two hypothesis studied in this work, the SCR and the MCP.

It can be argued that the longitudinal changes observed in the DOC and FDOM of the different size fractions could partially be due to mixing of LIW with other water masses instead of ageing. However, water mass mixing would reinforce our hypothesis since LIW in the western basin is the oldest water mass of the whole Mediterranean Sea. Then, if LIW is mixing with the surrounding water masses, which are younger than LIW, the longitudinal changes in DOC and its partition in aLMW and aHMW compounds should be more evident than those really observed.

In summary, we provide *in situ* support for the SRC hypothesis, out of the artefacts of microbial incubation experiments. Our results are neither distorted by alterations in the structural continuum of DOM during size-fractionation previous to *in vitro* experiments nor by subsequent “bottle effects” during incubations. Fractionation by ultrafiltration before *in vitro* degradation experiments breaks down the DOM structural continuum and, therefore, could not be the most appropriate approach for studying the DOM size-reactivity. Although our study is based on a relatively small and shallow overturning cell, we believe that our results can be extrapolated to the global ocean. To confirm this, it would be necessary to perform similar studies in the main water mass formation areas of the world ocean.

## Methods

### Study area and sampling

The main water masses observed in the Mediterranean Sea are the Atlantic water (AW) in the epipelagic layer, the LIW in the mesopelagic layer and the Eastern (EMDW) and Western (WMDW) Mediterranean Deep waters in the bathypelagic layer. The Atlantic inflow enters the Strait of Gibraltar as a surface current of salinity about 36.5, being slightly modified through mixing with the outflowing Mediterranean waters. This Modified Atlantic Water (MAW) moves towards the East as part of an overturning cell that involves the whole Mediterranean Sea and leads to the formation of intermediate waters in the eastern basin^24, 25, 46^.

Water samples were collected at nine stations (solid red circles in Fig. 1) during the trans-Mediterranean cruise HOTMIX aboard the R/V Sarmiento de Gamboa in the spring of 2014 (Heraklion, Crete, 27^th^ April – Las Palmas, Canary Islands, 29^th^ May). At each station, full-depth continuous conductivity-temperature-depth (SBE 911 plus CTD probe), dissolved oxygen (SBE-43 oxygen sensor) and chlorophyll fluorescence (SeaPoint fluorometer) profiles were recorded. These probes were attached to a rosette sampler (SBE 38) equipped with 24 Niskin bottles of 12 litres. The temperature and pressure sensors were calibrated at the Sea Bird laboratory before the cruise. Water samples were collected to analyse salinity (S), dissolved oxygen (DO) and chlorophyll *a* (Chl *a*), data used to calibrate the sensors for conductivity, DO and fluorescence, respectively. Conductivity measurements were converted into practical salinity scale values^47^. Samples for salinity were collected and stored in 250 mL type II glass and measured with a Guildline Portasal salinometer Model 8410A. Chl *a* concentration was determined in seawater samples (500 mL) filtered through Whatman GF/F filters and stored frozen until analysis. Pigments were extracted in cold acetone (90% v/v) for 24 h and analysed by means of a 10 AU Turner Designs bench fluorometer, previously calibrated with pure Chl *a* (Sigma Aldrich)^48^. Dissolved oxygen samples were taken in pyrex “iodine titration” flasks with flared necks and ground glass stoppers, with a nominal volume of about 115 mL. Dissolved oxygen was determined following the modified Winkler potentiometric method^49^. The apparent oxygen utilization, (AOU = O_2_sat – O_2_) was calculated using a algorithm for the oxygen saturation^50^, O_2_sat. Four to five depths were sampled depending on the bathymetry of the stations, except for the station at the Strait of Sicily (stn 13) where only LIW was sampled due to its shallowness. Deep chlorophyll maximum (DCM) samples were distinguish according to the maximum fluorescence intensity, the LIW was sampled at the absolute maximum of the salinity profile of each station, the oxygen minimum layer (OML) was established on basis of the absolute minimum of the dissolved oxygen profile, and the deep waters were sampled according to the salinity and temperature characteristic of the bathypelagic zone of the eastern and western Mediterranean basins (Fig. S1).

### DOM size-fractionation

DOM water samples were collected in 5-litres acid-cleaned polycarbonate carboys and stored in the dark at 13 °C until filtration within 5 hours. Filtration was performed through precombusted (450 °C, 4 h) Whatman GF/F filters in an acid-clean all-glass filtration system under positive pressure with low flow of high purity N_2_. Two-litre aliquots of the filtrate were collected in acid-cleaned PTFE bottles for DOM size-fractionation using an ultrafiltration cell (Millipore, 2000) equipped with a cut-off membrane of 1000 Da (Millipore, PLAC 150 mm). A pressure of 55 psi, using high purity N_2_, was maintained during the fractionation splitting the DOM into an apparent high molecular weight (aHMW, >1000 Da; 0.5 L) and an apparent low molecular weight (aLMW, <1000 Da; 1.5 L) fractions. The ultrafiltration cell was cleaned between samples by passing 0.3 L of NaOH through the membrane filter, followed by three rinses with 1 L of MQ water. The efficiency of the ultrafiltration cell (>90%) was checked using a solution of vitamin B12 (1355 Da; Sigma, 50 mg L^−1^). For large-volume ultrafiltration systems, previous works^29, 51^ reported lower efficiency rates (about 80%) using the same solution of vitamin B12. This fact was due to the general decrease of the retention rate with increasing concentration factors (CF). In our case, the CF (sample volume/retentate volume) was 4. We used a low CF for two main reasons: i) the ultrafiltration time increased with higher CF, so we keep it low in order to processes a significant number of samples, and ii) to ensure a pure aLMW fraction, which is much less studied compared to the aHMW fraction. Note that due to this CF and the ultrafiltration cell system used in this study, up to 25% of the aHMW fraction may correspond with low molecular weight molecules. In this system the sample is not recirculated, but it is being forced to a continuous and vigorous stirring inside the ultrafiltration cell. Then, the bulk DOM and aLMW fraction are pure treatments, but the aHMW fraction presents all the high molecular weight molecules (100%) plus a portion of the low molecular weight molecules (up to 25% of the aHMW fraction) due to the retention of aLMW-DOM during ultrafiltration. Taking into account that the %aHMW ranged between 76 to 55% and that in our ultrafiltration system the retentate was 0.5 L and the permeate 1.5 L, applying a simple system of 2 linear equations with 2 unknowns, the lower molecular weight molecules in the aHMW fraction varied between 8 and 15%, respectively.

### Dissolved organic carbon (DOC) and fluorescence spectroscopy (FDOM)

Approximately 100 mL of the DOM filtrate, and the HMW and LMW fractions were collected for DOC and FDOM analyses. Aliquots of 10 mL were collected in precombusted (450 °C, 12 h) glass ampoules for DOC determination. These samples were acidified with H_3_PO_4_ (85%, p.a., Merck) to pH < 2 and the ampoules were heat-sealed and stored in the dark at 4 °C until analysis in the base laboratory. DOC concentrations were measured using a Shimadzu TOC-V organic carbon analyser following a high temperature catalytic oxidation (HTCO) method. The system was calibrated daily with potassium hydrogen phthalate (99.95–100.05%, p.a., Merck). The precision of the equipment was ± 1 μmol L^−1^. The accuracy was successfully tested daily with the DOC reference materials provided by D. A. Hansell (University of Miami, USA). FDOM was determined on board with a Perkin Elmer LS55 luminescence spectrometer. Slit widths were 10.0 nm for both excitation and emission wavelengths. Measurements were performed at constant room temperature (25 °C) in a 1 cm quartz fluorescence cell and Milli-Q water was used as a reference blank. The spectrofluorometer was tested daily: the intensity of the Raman peak was evaluated using a sealed Milli-Q water cell (Perkin Elmer) while p-terphenyl and tetraphenylbutadiene methacrylate blocks (Starna) were employed to check the instrument signal intensity at the excitation-emission wavelengths characteristic of the aromatic amino acids and humic-like substances, respectively. Measurements were performed at the classical fluorescence peaks^18^, selecting Ex/Em wavelengths of 340/440 nm (peak C), due to humic substances of terrestrial origin, and 280/350 nm (peak T), due to protein-like substances. The fluorescence of each peak was determined by subtracting the Milli-Q blank peak height from the sample average peak height. All the samples were normalized using a solution of quinine sulphate dihydrate (≥99.0%, purum for fluorescence, Fluka) and tryptophan (≥99.0%, Fluka) standards in H_2_SO_4_ 0.05 M (95–97%, p.a., Merck) allowing to express fluorescence in normalized fluorescence intensity units (NFIU^52^).

## Electronic supplementary material

Supplementary Information
