# Supplementary material for: Deep-ocean dissolved organic matter reactivity along the Mediterranean Sea: does size matter?
Source: Sci Rep. 2017 Jul 18;7:5687. doi: 10.1038/s41598-017-05941-6 (PMC5515921; doi:10.1038/s41598-017-05941-6)

Supplementary information

Deep-ocean dissolved organic matter reactivity along the Mediterranean Sea: does size matter?

Alba María Martínez-Pérez[a,]*, Xosé Antón Álvarez-Salgado[a], Javier Arístegui[b] & Mar Nieto-Cid[a]

[a] Consejo Superior de Investigaciones Científicas - Instituto de Investigacións Mariñas (CSIC-IIM), Vigo, Spain

[b] Instituto de Oceanografía y Cambio Global (IOCAG), Universidad de Las Palmas de Gran Canaria, 35017 Las Palmas de Gran Canaria, Spain.

*Corresponding author: albam@iim.csic.es

Additional DOM size-fractionation considerations

Apparent oxygen utilization (AOU) across the Mediterranean Sea showed mean ± SD values of 4.5 ± 3.1, 50.2 ± 30.3 and 64.4 ± 5.2 µmol kg^{-1} for the deep chlorophyll maximum (DCM), Levantine Intermediate Water (LIW) and deep water samples, respectively. The corresponding SD values translated into carbon equivalents using the canonical Redfield $-O_2/C$ ratio of 1.4, AOU-Ceq, were 2.2, 21.6 and 3.7 µmol-C kg^{-1} for DCM, LIW and deep waters, respectively. Therefore, the core-of-flow of the LIW is the layer that presents the largest variability of AOU-Ceq, an order of magnitude above the DCM and deep layers.

At the DCM there is not a clear relationship between AOU and the bulk (DOM), apparent high (aHMW) and apparent low (aLMW) molecular weight fractions of dissolved organic matter. This is an expected result given the narrow ranges of variability of AOU-Ceq and dissolved organic carbon (DOC). In this regard, DOC at the DCM was 64.3 ± 2.8, 41.9 ± 5.5 and 23.0 ± 5.3 µmol-C L^{-1} for bulk DOM, aHMW and aLMW fractions, respectively (circles in Fig. S2A, B, C). It is noticeable too that the SD of DOC, 2.8 µmol-C L^{-1}, was larger than the SD of AOU-Ceq, 2.2 µmol-C kg^{-1} suggesting that this behaviour could be related to the primary production processes dominating in this layer, which lead to lower AOU and higher DOC values.

In the deep waters (squares in Fig. S2A, B, C) mean ± SD concentrations of DOC were 42.6 ± 1.9, 28.7 ± 2.7 and 16.3 ± 2.5 µmol-C L^{-1}, for bulk DOM, aHMW and aLMW fractions, respectively. This range is also quite narrow considering that we are merging all samples from more than 1000 m in either the eastern and western basin. In this case, the SD of AOU-Ceq for the deep waters, 3.7 µmol-C kg^{-1}, was about 50% larger than the SD of DOC, indicating that DOC should be a major contributor to the oxygen demand of the deep water from their respective formation site to the study zone.

The samples collected at the salinity maximum, corresponding to the higher proportion of LIW (triangles in Fig. S2A, B, C), presented the highest AOU variability, with lower values at the stations located near the formation area (Levantine basin) and higher in the western basin. Moreover, these intermediate waters presented wide DOC changes, (mean ± SD) except for the LMW fraction, (53.2 ± 6.4, 34.1 ± 7.1 and 20.4 ± 2.0. µmol-C L^{-1} for bulk DOM, aHMW and aLMW, respectively) associated with the DOM mineralization processes taking place in this layer along its route westwards (AOU increment).

This size-fractionation pattern is also observed when plotting AOU against the optical properties of DOM. The carbon-specific fluorescence intensity of humic-like compounds (peak C*; Fig. S2D, E, F), considered as a proxy to microbial degradation processes[1-2], showed higher fluorescence intensity per carbon unit for the aLMW fraction for all samples collected at different depths (DCM, Deep and LIW). In addition, in general terms this fluorescence intensity was significantly higher (p-value < 0.001) in the western basin, due to the higher heterotrophic activity in this area[3]. Specifically, in the deep waters a slight increase of peak C* with the AOU was observed for the aLMW fraction (Fig. S2F), indicative of the generation of humic-like substances in this fraction. However, it was not observed for the bulk DOM, which means that the rate of peak C production in the LMW fraction is higher than the DOC consumption rate. Analogously, the carbon-specific fluorescence intensity of protein-like compounds (peak T*; Fig. S2G, H, I), considered as a proxy for bioavailable substances, showed the same trend of higher fluorescence intensity per carbon unit for the LMW fraction.

References

1. Nieto-Cid, M., Álvarez-Salgado, X. A. & Pérez, F. F. Microbial and photochemical reactivity of fluorescent dissolved organic matter in a coastal upwelling system. *Limnol. Oceanogr.* **51,** 1391–1400 (2006).

2. Lønborg, C., Álvarez-Salgado, X. A., Martínez-García, S., Miller, A. E. J. & Teira, E. Stoichiometry of dissolved organic matter and the kinetics of its microbial degradation in a coastal upwelling system. *Aquat. Microb. Ecol.* **58,** 117–126 (2010).

3. Luna, G.M., Bianchelli, S., Decembrini, F., De Domenico, E., Danovaro, R., & Dell'Anno, A. The dark portion of the Mediterranean Sea is a bioreactor of organic matter cycling. *Global Biogeoche.m Cycles* **26**(2) (2012).

Figure S1: Hydrography of the Mediterranean Sea. (A) Salinity (S), (B) potential temperature (θ) in ºC, (C) apparent oxygen utilization (AOU) in µmol kg^{-1} and (D) fluorescence of chlorophyll *a* (Chl *a*) in mg m^{-3} obtained from the sensors attached to the rosette sampler. Black, yellow, green and red circles represent samples collected in the epipelagic layer (DCM samples), Levantine Intermediate Water (LIW), oxygen minimum layer and deep waters, respectively. The dashed black line represents the route of the LIW along the transect. Note that the depth is displayed in a non-linear scale. Figure created using Ocean Data View (Schlitzer, R., Ocean Data View, odv.awi.de, 2017).

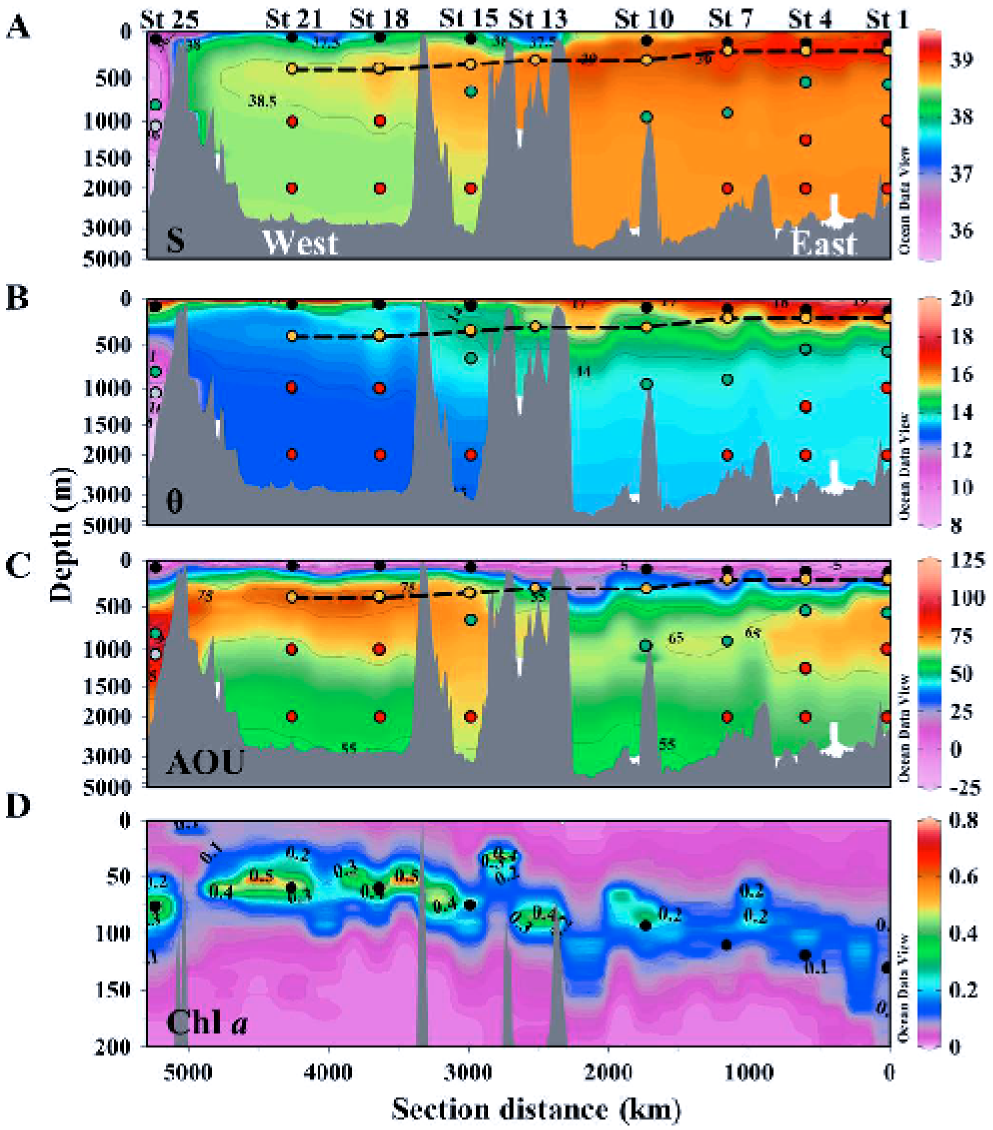

Figure S2: Range of variability of DOC and FDOM in DOM, apparent LMW DOM and apparent HMW DOM with respect to AOU for all collected samples. (A-C) DOC, (D-F) humic-like fluorescence of the peak C per carbon unit (peak C*) and (G-I) protein-like fluorescence of the peak T per carbon unit (peak T*). Black filled circles, black circles, black filled triangles, black triangles, black filled squares and black squares represent samples collected at the DCM in the East, DCM in the West, LIW in the East, LIW in the West, deep samples in the East and deep samples in the West, respectively. The blue rectangles cover the DCM and deep samples and the red ones the LIW samples. Error bars represent standard errors.

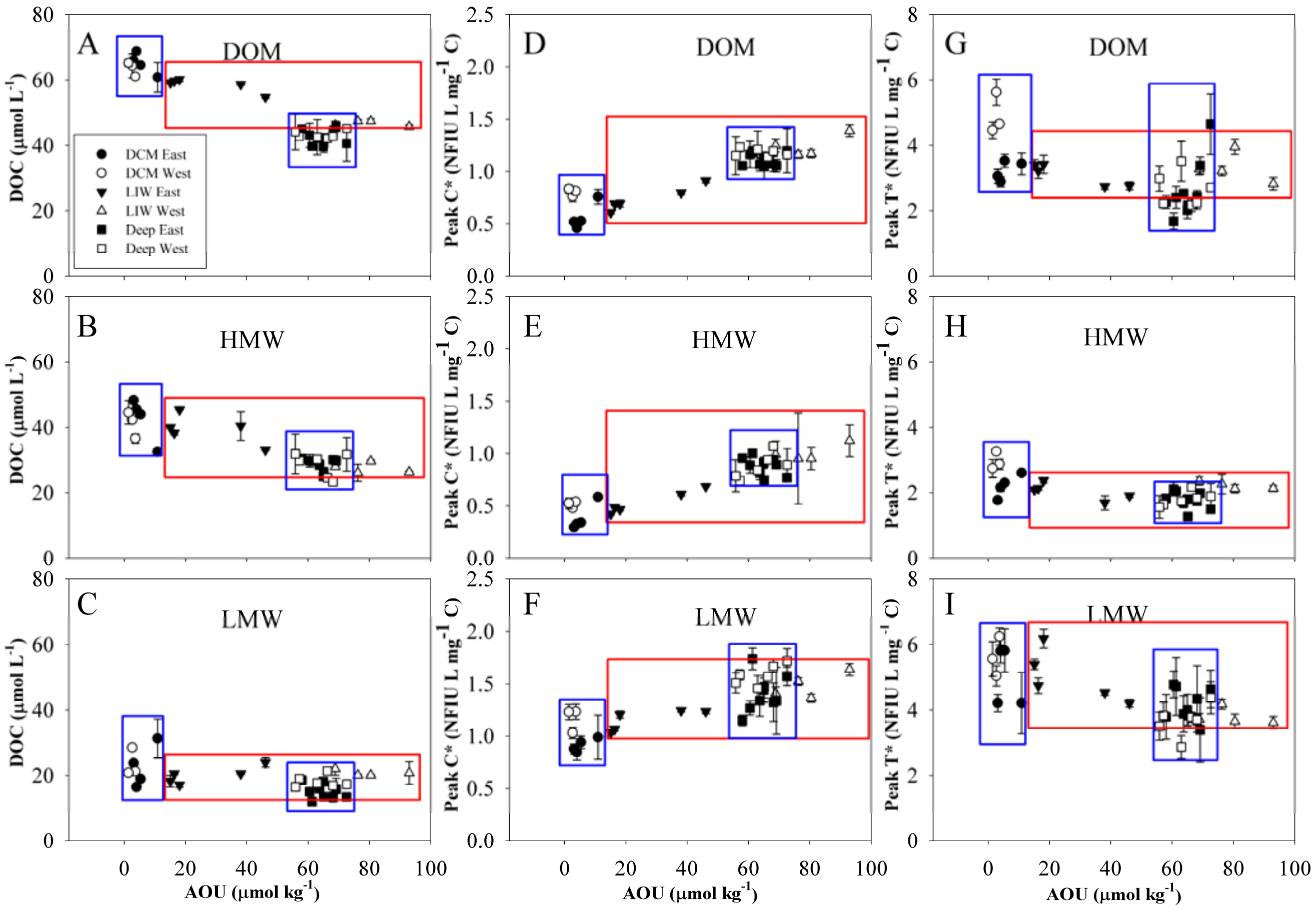

Supplement: Supplementary file 1 — Supplementary Information [file 41598_2017_5941_MOESM1_ESM.pdf]
